# Education of research ethics for clinical investigators with Moodle tool

**DOI:** 10.1186/1472-6939-14-53

**Published:** 2013-12-12

**Authors:** Arja Halkoaho, Mari Matveinen, Ville Leinonen, Kirsi Luoto, Tapani Keränen

**Affiliations:** 1 Research Ethics Committee/Science Service Center, Kuopio University Hospital, P.O BOX 100, Kuopio FI-70029 KYS, Finland; 2 Science Service Center, Kuopio University Hospital, P.O BOX 100, Kuopio FI-70029, KYS, Finland; 3 Department of Neurosurgery, Kuopio University Hospital, Kuopio, Finland; 4 Institute of Clinical Medicine University of Eastern Finland, Kuopio, Finland; 5 School of Pharmacy, Faculty of Health Sciences, University of Eastern Finland, Kuopio, Finland; 6 Department of Neurology, Kanta-Häme Central Hospital, Hämeenlinna, Finland

**Keywords:** Bioethics, Research ethics, Web learning, Legislation

## Abstract

**Background:**

In clinical research scientific, legal as well as ethical aspects are important. It is well known that clinical investigators at university hospitals have to undertake their PhD-studies alongside their daily work and reconciling work and study can be challenging. The aim of this project was to create a web based course in clinical research bioethics (5 credits) and to examine whether the method is suitable for teaching bioethics. The course comprised of six modules: an initial examination (to assess knowledge in bioethics), information on research legislation, obtaining permissions from authorities, writing an essay on research ethics, preparing one’s own study protocol, and a final exam. All assignments were designed with an idea of supporting students to reflect on their learning with their own research.

**Methods:**

57 PhD-students (medical, nursing and dental sciences) enrolled and 46 completed the course. Course evaluation was done using a questionnaire. The response rate was 78%. Data were analyzed using quantitative methods and qualitative content analysis.

**Results:**

The course was viewed as useful and technically easy to perform. Students were pleased with the guidance offered. Personal feedback from teachers about students’ own performance was seen advantageous and helped them to appreciate how these aspects could be applied their own studies. The course was also considered valuable for future research projects.

**Conclusions:**

Ethical issues and legislation of clinical research can be understood more easily when students can reflect the principles upon their own research project. Web based teaching environment is a feasible learning method for clinical investigators.

## Background

Clinical research is associated with several important ethical and legal issues. The research subjects are often patients in need of care and thus, the protection of research subjects must be a primary concern. This is especially important in vulnerable subjects such as children and other individuals who themselves cannot provide informed consent. Despite globally established ethical guidelines such the Helsinki Declaration [1], fraud and misconduct are still a problem in research [2]. In Finland, a single law, Medical Research Act [3], covers all areas of medical research, but within the European Union, the situation appears to be much more variable [4]. Thus, clinical investigators need to possess knowledge and understanding of ethical principles as well as relevant legislation which they should able to apply in their research.

In the United States, it is mandatory that research institutions receiving funds from the Public Health Service should provide education in the responsible conduct of research for all their research personnel [5]. To this end, traditional contact teaching courses as well as programs utilizing online methods have been developed to meet the needs for training in research ethics [6-9]. Within the EU, approaches in teaching research ethics vary from country to country [10]. It has been noted that to ensure that it is relevant, the education in medical ethics, it must emphasize the link between ethics and clinical practice [11].

Training in research ethics has many goals but also faces many challenges [12]. One important issue is that clinical investigators, especially those at the beginning of their scientific careers, often are confronted by dilemmas in reconciling their clinical duties with their research activities [13]. Today web-based methods are being increasingly used in education on practice and ethics of clinical research [14-16]. Web-based learning offers a feasible means for studying alongside daily work duties. The content of the teaching material is usually composed of audio or videotapes with links to other educational material. A large number of students can take part in the course and they can either discuss with each other in groups or with their tutors. Learning material may also involve confidential issues and, thus an appropriate level of confidentiality needs to be assured [17]. The Moodle learning environment, one of the widely used web based learning tools, has been proved to be useful for provision of courses in universities, also in the area of medical education [15,16]. In fact, web based learning continues to increase, but surprisingly, web based courses on research ethics are still rather uncommon [7,8,18]. The aim of this project was first to develop a web based course in clinical research bioethics (5 study credits) and then to determine out whether the method would be suitable for teaching bioethics for PhD students working in the field of clinical research [9].

## Methods

### Description of the clinical research moodle course

The content of the course was constructed by several experts in bioethics and clinical research in the Science Service Center of Kuopio University Hospital. A pilot version of the course was tested and evaluated by seven clinical research experts and three PhD students [9].

The course comprises of six modules: 1) initial examination, 2) information on research legislation and international declarations 3) obtaining approvals from national and international authorities, 4) writing an essay on research ethics based on the ethical principles to be applied in clinical research, 5) preparing one’s own study protocol and 6) a final examination. A summary of the modules is presented in Table 1.

**Table 1:** Summary of the course modules

**Modules with content**	**Form of assignment**	**Evaluation**
**Initial examination: Basic knowledge in bioethics and legislation related to clinical research**	Multiple-choice questions	Accept/fail
• Knowledge about basic principles in bioethics		
• Knowledge of the Helsinki Declaration		
• Knowledge of the Finnish legislation		
• Principles of Good Clinical Practice		
**Information on research legislation and international declarations**	Essay	Individual feedback from tutor, accept/fail
• National legislation regarding clinical research		
• The Helsinki Declaration		
• Principles of Good Clinical Practice		
**Approvals from national authorities for different types of research**	Essay	Individual feedback from tutor, accept/fail
• Clinical drug trials		
• Clinical trials on medical device		
• Tissue research		
• Register based study		
• Studies with GMM (genetically modified micro-organisms)		
**Research ethics**	Essay	Individual feedback from tutor, accep/fail
• Knowledge of the basic principles of bioethics:		
• basic ethical principles: respect for life and autonomy,		
• beneficence, avoiding harm, equitable selection of subjects, privacy and justice		
• risk/benefit assessment		
• elements of informed consent		
• privacy and confidentiality		
• integrity in science		
**Preparing own study protocol**	Written study protocol (5–10 pages) summary of the previous assignments	Individual feedback from tutor, accept/fail
**Final examination**	29 general multiple-choice question	Correct answer gives a point and false remove. Three trials.
• Knowledge of basic principles in bioethics		
• Knowledge of the Helsinki Declaration		
• Knowledge of the Finnish legislations		
• Principles of Good Clinical Practice		
• Knowledge of national authorities for different research type		

All assignments, with the exception of the final examination, were designed with an idea of encouraging students to reflect their own research in their learning. The course was designed to be completed in three months and students could proceed at their own pace. Table 2 describes planned course actions and predicted time for completion of each activity.

**Table 2:** Course actions and the average time designed for taking the course

**Actions for students**	**Time designed for studying (hours)**
**1. Orientation**	**5 h**
• Student familiarizes herself/himself with the instructions, content, objectives and assignments	3 h
• Student sets her/his objectives for the course and plans the use of time	1 h
• Student familiarizes herself/himself with the use of Moodle learning environment and the instructions	1 h
**2. Knowledge building**	**83 h**
• Student searches materials related to the subject (literature, websites, articles)	5 h
• Student familiarizes herself/himself with the course materials (video lectures, written materials, reference lists and links to relevant materials) and understands the central concepts and contents of the course	52 h
• Performs out the course assignments (4 assignments/6,5 h)	26 h
**3. Learning outcomes assessment and course evaluation**	**50 h**
• Student takes the self-evaluation tests (initial and final tests)	1 h
• Student writes study protocol (summary of all assignments)	48 h
• Student provides feedback on the course	1 h
**Total**	**138 h = 5 credits**

Altogether 5 tutors, representing expertise in various areas of medicine, nursing sciences and bioethics, were recruited into the course. The tutors were neither supervisors of the participating student nor members of the study group in question. The tutors evaluated the assignments and provided written feedback. All communication between students and the tutors was conducted in the Moodle environment. The estimated time for each student with all assignments was 60–240 minutes per tutor. In order to ensure confidentiality of the research plans, the course did not include any interactions or discussions between the students; only the tutors could view the returned assignments. Each assignment was directed to a selected tutor who had a PhD degree and competence in the scientific field of the student as well as knowledge in the legal or ethical aspects of human research. The course coordinator was responsible for practical issues including technical guidance and reminding students about course deadlines.

### Participants

The course was offered to doctoral (PhD) students of the principal clients of the Science Service Center, the University of Eastern Finland and the Kuopio University Hospital. All the students, representing various fields of medicine, dentistry and nursing sciences, needed to have a study project involving clinical research in humans. Altogether 57 PhD-students were enrolled and 46 of them completed the course.

### Outcome measures and data analysis

After completion of the course, each student had to pass a web based examination which included a total of 29 general multiple choice questions about principles in bioethics, human research legislation and specific declarations and approvals from relevant authorities (see Table 1).

After finishing the course, the students completed a course evaluation form delivered via the Moodle environment in order to ensure anonymity. The form collected the opinions and overall satisfaction of the students with the course as a learning tool in bioethics [8]. The questionnaire included 13 items and the answers were rated using a 5-point Likert scale (1 do not agree - 5 totally agree). Additionally, five open ended questions were included to provide the possibility for written feedback about the course in general, the training material and assignments, work of tutors, and developmental ideas for the future. The response rate was 78%. Data were analyzed using the Moodle statistics tool. Mean values are shown. Open ended questions were analyzed using content analysis and the answers were grouped within the themes. The groups were used to identify elements that described the data, and the concepts with similar content were combined to form upper concepts. The results were presented as quotes to highlight the significance that the participants allotted to the theme being questioned [19,20].

### Ethical consideration

According to the Finnish Research Act [3], an opinion from a research ethics committee is not required for this type of research. Anonymous questionnaire, without any possibility for participants’ identification, about course feedback cannot be regarded to provide sensitive, potentially harmful information about the participants. Participants were aware that participation in the questionnaire was voluntary and returning of the questionnaire was regarded as consent.

## Results

All of the students who completed the course actually passed the final examination. In general, students were extremely content with the course. The mean score for all questions was remarkably good, the lowest was 3.95 and the highest 4.84 (Table 3).

**Table 3:** Students’ opinions about the course and the course content (means)

**Questions**	**Mean (Scale: 1 do not agree - 5 totally agree)**
The course objectives were clearly stated.	4.29
The course met the objectives set for it	4.34
The practical arrangements for the course were good.	4.32
The communication between tutors and persons in charge worked well during the course.	4.61
The work required in the course is appropriate for the credit offered.	4.24
I was provided enough guidance for carrying out the course assignments.	4.24
I was provided with enough guidance on the allocation of my time in the course.	3.95
I was provided with enough guidance about using the learning environment.	3.97
I learned new issues.	4.61
The course enhanced my understanding of my own research project.	4.71
I will be able to apply the information and knowledge gained in the course in future.	4.84

In the open ended questions (Table 4) more specific information was inquired. The course was considered useful and technically straightforward to perform. The students stated that the course was well planned with clear objectives. The education material e.g. links to the legislation and videos were considered as being most useful. Furthermore the course structure, and how the material was presented, were both rated as advantageous. The textual material was easy to read.

**Table 4:** Opinions of the participants

**Themes**	**N**	**Examples of simplified quotes**
Feedback on the material	28	“Web based material was very good and also videos were well structured.”
		“Reading the material was laborious but rewarding. The video lectures helped me to understand the statutes.”
		“There was a lot of material and it was well outlined as well as flexibly at hand.”
Feedback from the assignments	29	“Assignments were explicit and useful for my own research.”
		“Sometimes I considered assignments little like nit-picking but on the other hand, I did not have enough time for all of them because short and vague answers were not sufficient.”
		“Quite challenging, a lot to think about.”
Feedback to tutors	26	“Special thanks for the tutors for their prompt comments so that I could move on.”
		“All feedback was really meaningful.”
		“Thanks for the support and reminders.”
Positive issues	28	“Practicality”
		“As a whole, the support given for the scientist was valuable when it comes to planning and performing the study.”
		“Self-directed studying at a convenient time and a possibility to revise through the study material.”
Development	20	The goals of initial and final examinations? Some parts of those tests were not relevant to my research.”
		“More video lectures.”
		“Some overlap in assignments.”

The students considered the course as being valuable for their current as well as for the future research projects. They reported that the course had increased their knowledge of the legal and ethical issues surrounding of clinical research. Some students found it challenging to evaluate the implications for their own project in every assignment, but in the end, this was regarded important. Some of them pointed out that it was difficult to find the time to complete the course. In addition, some students had a more challenging research frame which required for more time and effort.

Students were satisfied with the support, guidance and teaching provided by the tutors. Personal feedback from the tutors about the research project of the student was considered as advantageous and it helped to increase their understanding of different aspects of research.

The main advantage, which was often pointed out by the students, was the possibility to complete the course at their own pace. The students proposed that in the future this kind of course should be mandatory for all scientists in the field of clinical research. The possibility to focus in the assignments on the student’s own research project was considered as useful and it was recommended that this strategy should be continued in the future. In addition, the consideration of ethical aspects in their essays, particularly the requirement to apply ethical principles in the description of research processes e.g. obtaining informed consent, was considered positive and this had expanded their understanding of the importance of ethics in research.

## Discussion

Our findings indicate that web based teaching is a feasible way to provide training on research ethics for clinical investigators. Previous programs using the internet in research ethics education have utilized the web mainly as a source of teaching material [7,8]. Our program combines the availability of video lectures and other learning material with web-based interaction between students and tutors. Previous studies have found that on-line and on-site training result in rather similar improvements in the knowledge of research ethics [9,18]. Because web-based education, especially the Moodle environment, offers several advantages over on-site teaching for professionals, it may represent a very feasible method for education in research ethics. Especially for the health science investigators, busy with their clinical duties, on-line teaching may represent a flexible means for education [21]. Furthermore, although our course was offered to scientists from one university and hospital, on-line teaching can reach students from a wide geographical area [18].

Overall, students in our program well as those of Aggarwall et al. [18], were very satisfied with the web based education. Furthermore, all of our enrolled students also completed the course. Drop-out rate was also very small among the on-line students of Aggarwall et al. [18] Feedback from the students suggests that our course met well its goals and objectives and that Moodle based learning was feasible. Students appreciated the availability of the course materials, the ease of using the Moodle tool and the possibility to perform studies at their own pace and chosen time. Previously, these have been emphasized as benefits associated with web based learning [14,22]. Aggarwall and coworkers [18] also reported that in their ethics course on-line students were less likely than the on-site students to consider the progress of the course as being too fast.

Our findings suggest that an appreciation and adoption of ethical issues and legislation of clinical research can be beneficial to allow students to reflect on how the principles apply to their own research project, e.g. recognize important ethical issues in the protection of study subjects. We consider that a major advantage of our course was the availability of on-line tutoring of the students. The tutors, experts in research as well as educated in ethics, were able to recognize individual learning needs of the student and provide immediate feedback. However, a major challenge in this kind of Moodle teaching is to be able to recruit tutors who can commit themselves to the course for a prolonged period of time.

One of the possible limitations of our course is the lack of possibilities to conduct any group discussions or communication between the students through the internet and seminars. The opportunity to share experiences with different research related issues might increase the appreciation of the multidimensional nature of bioethics [9]. The inclusion of a discussion portal into our course program will be considered in the future. Although the response rate in the course evaluation was high, the rather small total sample size must be considered. Furthermore, our study principally assessed the opinions and attitudes of the students concerning the course. Since all the students were able to pass the final examination, the course clearly met its teaching goals. However, it must be emphasized that research ethics cannot be mastered by taking a single course. The intention is to stress that research ethics are a life-long and an ongoing process.

## Conclusion

In conclusion, our program, which involves a combination of web based learning material and interaction between students and their tutors, is a feasible method for teaching research ethics.

### Ethical approval

According to the Finnish Research Act [2], an opinion from a research ethics committee is not required for this type of research. Anonymous questionnaire, without any possibility for participants’ identification, about course feedback cannot be regarded to provide sensitive, potentially harmful information about participants. Participants were aware that participation in the questionnaire was voluntary and returning of the questionnaire was regarded as consent.

## Competing interests

The authors declare that they have no competing interests.

## Authors’ contributions

AH and TK designed the manuscript. AH analyzed the data. KL, AH, VL and TK designed the course and were as tutors. AH, MM and TK wrote the manuscript. KL and VL commented the paper. All authors contributed to the significant revision of the paper and approved the final manuscript for publication.

## Pre-publication history

The pre-publication history for this paper can be accessed here:

http://www.biomedcentral.com/1472-6939/14/53/prepub
